# Electrochemical studies on *Cocos nucifera* (coconut hair oil) derived carbon soot as an electrode material for EDLC application using non-aqueous NaPF_6_ electrolyte

**DOI:** 10.1038/s41598-026-42749-9

**Published:** 2026-03-04

**Authors:** Anurag Tyagi, Rita Kumari, Rekha Gupta, Chhaya Ravi Kant, Kuldeep Mishra

**Affiliations:** 1 Noida Institute of Engineering & Technology, Greater Noida, G.B. Nagar, Affiliated to Dr. A. P. J. Abdul Kalam Technical University, Lucknow, Uttar Pradesh India; 2 Indira Gandhi Delhi Technological University for Women, New Delhi, Delhi India; 3 Symbosis institute of Technology, Symbiosis International (Deemed University), Pune Campus, Pune, 412115 India

**Keywords:** *Cocos nucifera*, Flame synthesis method, Carbon soot, EDLC, Specific capacitance, Electrochemical properties, Chemistry, Energy science and technology, Materials science

## Abstract

*Cocos nucifera* (Coconut hair oil) was burned using wick-and-oil technique (gas-phase combustion) known as flame synthesis method to obtain carbon soot for electrode material. Synthesized carbon soot was chemically activated using optimized ratio of activating agents ZnCl_2_ (1:1, wt. /wt.) and KOH (1:2, wt./wt.), followed by thermal treatment at 900 °C. XRD analysis showed that the coconut oil-derived carbon soot (CoCS) has a crystallite size of ~ 1.92 nm with an interlayer spacing of 3.62 Å. After chemical activation, the crystallite size slightly altered only, while a small increase in interlayer spacing was observed (3.65 Å for ZnCl₂ activation and 3.71 Å for KOH activation), revealing subtle structural modification of the carbon framework. SEM analysis revealed a well-connected microporous network with reduced agglomerate size after activation, while EDAX confirmed increased carbon content following chemical treatment. BET results showed a substantially enhanced surface area and mesoporous structure of the KOH-activated CoCS, leading to favorable pathways for electrolyte ion transport. Among the studied samples, KOH-activated CoCS demonstrated the best electrochemical performance, delivering a specific capacitance of ~ 176 F g⁻¹, with an energy density of ~ 6.11 Wh kg⁻¹ and a maximum power density of ~ 395 W kg⁻¹. The simple synthesis route, favorable structural characteristics, and competitive electrochemical performance highlight the potential of coconut oil-derived carbon soot as a scalable and sustainable electrode material for EDLC and other energy storage applications.

## Introduction

 Addressing the global energy problems is a difficult task. It has been determined that a number of energy storage technologies, such as batteries, hybrid capacitors and supercapacitors may satisfy the energy requirement. Every energy storage technology has a unique set of benefits and drawbacks. For example, supercapacitor shows the high-power density with excellent cyclability along with low energy density. Supercapacitors are a breakthrough energy storage technology that presents unmatched advantages for environmentally friendly energy systems. Several efforts are underway to develop the supercapacitors in order to optimize the energy density. Due to the durability, quick response and compatibility, supercapacitor may create an efficient and environmentally friendly landscape. Their full potential will be unlocked by more research and development, bringing us one step closer to a low carbon, green and ecofriendly future powered by effective and adaptable energy storage technologies^1–3^. In recent years, machine learning has also been introduced as a potential tool for supercapacitor research. These data-driven approaches help in optimizing synthesis conditions and material properties for superior electrochemical performance, making material development faster and efficient than traditional trial-and-error methods. Such strategies are increasingly being used to guide the design of high-performance bio-derived and functionalized carbon materials for supercapacitor applications^4–7^. In addition to offering inexpensive and environmentally friendly options, these alternatives are essential for lowering greenhouse gas (GHG) emissions, accelerating the transition toward a sustainable future, and ensuring energy independence^8^. In order to fulfil the Sustainable Development Goals (SDGs), specifically SDG 13 (Climate Action) and SDG 7 (Affordable and Clean Energy) a clean, reliable, and low-cost energy must be made available to reduce the effect of climate change and greenhouse gas emission. Reusing waste material, such as carbon soot for electrode fabrication to store energy also encourages industrial innovation (SDG 9) by creating new energy storage technologies and supports sustainable consumption and production (SDG 12).

**Fig. 1:**
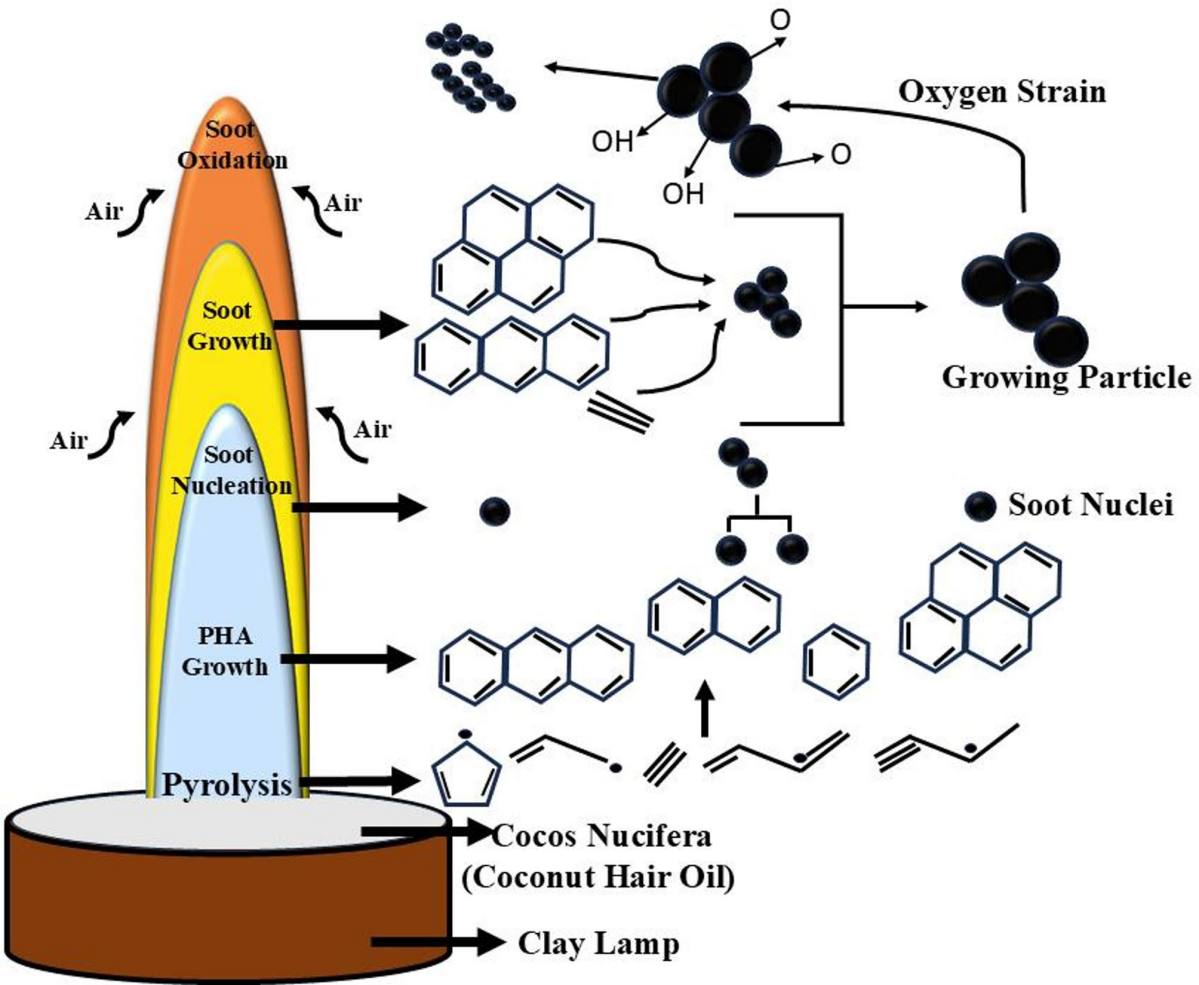
Schematic representation of the steps and processes involved in carbon soot formation during pyrolysis.

Carbon Soot (CS) contains particulate matters (PM), polycyclic aromatic hydrocarbons (PHAs) and other toxic elements. CS is a trending carbonaceous material, used to synthesize electrodes in supercapacitor application due to high surface area and mesoporous network^9^. In CS formation many processes and reactions take place during pyrolysis and combustion of fuels or precursor. These reactions and mechanism result the formation of many other carbon materials like Carbon Black, Carbon Nano Fibers (CNFs), fullerene and Carbon Nano Tubes (CNTs) under certain conditions^10–15^. During the combustion process under precise and specific environmental conditions, the growth of CS is initiated with quick chain reaction of hydrocarbons. Figure 1 represents the schematic diagram of different process involve in the carbon soot formation^16^.

Depending on the raw material synthesis technique and environment, the structural and chemical properties of the Carbon soot may be different. Flame synthesis method is a high temperature (1000˚C) combustion reaction for the soot formation. Gas-phase reactions take place during the combustion process and a condensed-phase molecular growth of typical 10–50 nm soot particle results at high temperature^17^. Agglomeration of series of spherical particles lead to formation of mature soot particles having a fractal geometry with a fractal dimension ~ 1.8 nm^18^.

Recent research primarily focusses on the synthesis of low-cost activated carbon with better porous architectures and increased storage capacity through structural and surface engineering for supercapacitor applications. Zhang et al.^19^ used the mixture of MnO_2_ and candle derived carbon soot to fabricated electrodes for EDLC device. This composite reveals the outstanding electrochemical performance having the capacitance ~ 309 F g^− 1^ at the current density 1 A g^− 1^ and excellent cycle life. D Mohapatra et al.^20^ synthesized carbon soot having layered structure by burning clarified butter and displayed the good capacitive behavior having specific capacitance ~ 102 F g^− 1^ with high charge discharge efficiency. V Sahu et al.^21^ represented the electrochemical investigations utilizing the mustard oil derived carbon aerogel shows specific capacitance of 102 F g^− 1^ at current density 0.5 A g^− 1^ while carbon aerogel with polyaniline (PANI) composite displayed improved capacity of 398.5 F g^− 1^. In another research, V Sahu et al.^22^ demonstrated that the composite of carbon soot with α-MnO_2_·0.3H_2_O displayed specific capacitance of 425 F g^− 1^ with excellent cyclability. Rita et al.^23^ synthesized carbon soot using soybean oil and waste engine oil with flame synthesis technique. Engine oil shows better electrochemical performance with maximum specific capacitance 61.4 F g^− 1^ while soyabean oil represented maximum capacitance of 44.8 Fg^− 1^ at current density 0.5 mA cm^− 2^. Waste frying oil and grape seed oil was also used for the synthesis of carbon nano onions (CNOs) shows good capacitive behavior with specific capacitance ~ 71 F g^− 1^ at 2 A g^− 1^ and ~ 54 F g^− 1^ at 0.1 A g^− 1^ respectively with significant cycle life^24,25^. The above reported research work used flame synthesis technique to synthesize carbon materials. During the activation process different gaseous environment was used such as CO_2_, N_2_ or any inert gases, which makes the activation process very complex and tedious and increases the synthesis cost.

In our previous studies, the wick-oil method (flame synthesis method) was used to synthesized carbon soot without any artificial environment. The activated CS (ACS) was produced using mustard oil followed by activation with ZnCl_2_ by Tyagi. et al.^26^. ACS electrodes represented good energy storage capacity of 50 F g^− 1^ at 0.5 mA cm^− 2^^26^. In another study by Tyagi et al., carbon soot was obtained using sinapis alba (yellow mustard oil) further activated with KOH (MoCS), this electrode material shows good capacitive behavior of 57.78 F g^− 1^ with good cycle life^27^. A sample of carbon soot using sesamum indicum oil (sesame oil) followed by chemical activation with ZnCl_2_ and KOH was also synthesized by Tyagi et al.^28^. ACS_K2_ represented superior electrochemical performance and durability with capacity of 94 F g^− 1^, energy density of 10.5 Wh kg^− 1^, power density ~ 1350 W kg^− 1^ and cycle life more than 2000 cycles.

This research work presents a low-priced, environmentally benign process for synthesizing the potential carbonaceous material utilizing *Cocos nucifera* (Coconut Hair Oil) in a precise air environment devoid of any non-biodegradable or harmful materials. This oil contains high saturated fatty acid, uniform combustion behavior, low intrinsic impurities and show consistent soot morphology, which qualify it as a stable reproducible carbon precursor. A flame-synthesis method is used to obtain the Coconut oil derived carbon soot (CoCS). The obtained CoCS was activated with two different activating agents, ZnCl_2_ and KOH, and the obtained activated CSs were investigated using different physical and electrochemical investigations. The structural and physical properties of synthesized CoCS, CoACS_ZnCl2_ and CoACS_KOH_ are characterized using X-ray photoelectron spectroscopy (XPS), X-ray diffraction (XRD), Brunauer-Emmett-Teller (BET) adsorption desorption, Fourier Transform Infrared Spectroscopy (FTIR) and Scanning electron microscopy (SEM). CoACS_ZnCl2_ and CoACS_KOH_ are used as an electrode material to fabricate electric double layer capacitor (EDLC) device to investigate electrochemical performance. In this research XRD peaks shows that carbon soot was successfully transformed into better crystalline domains after activation and exhibit electrochemical performance due to high specific capacitance, excellent rate capability and better cyclability. These properties positioned carbon soot as a strong electrode material for energy storage devices.

## Materials and methods

### Materials

*Cocos nucifera* (Coconut Hair Oil) was bought from neighborhood marketplace. Activating agent Potassium hydroxide (KOH) was procured from Finar, India. Other precursors including, Whatman glass microfiber filter paper of GF/D grade, for electrolyte preparation Ethylene carbonate (EC) (purity 98%), propylene carbonate (PC) (purity 99.7%), sodium hexafluorophosphate (NaPF_6_) (purity 98%), an adhesive binder poly (vinylidene fluoride-co-hexafluoropropylene) (PVDF-HFP) (MW ~ 4 × 10^5^), Zinc Chloride (ZnCl2) (purity ≥ 98%), KOH pellets (purity ≥ 85%), Hydrochloric acid (purity 37%) and Acetylene black of 99% metal basis with super P conductivity for conductive additive were procured from Sigma-Aldrich, USA. For current collector polished flexible graphite sheets were purchased from Toray, Japan.

### Synthesis of *Cocos nucifera* (coconut hair oil) (CoCS)

**Fig. 2:**
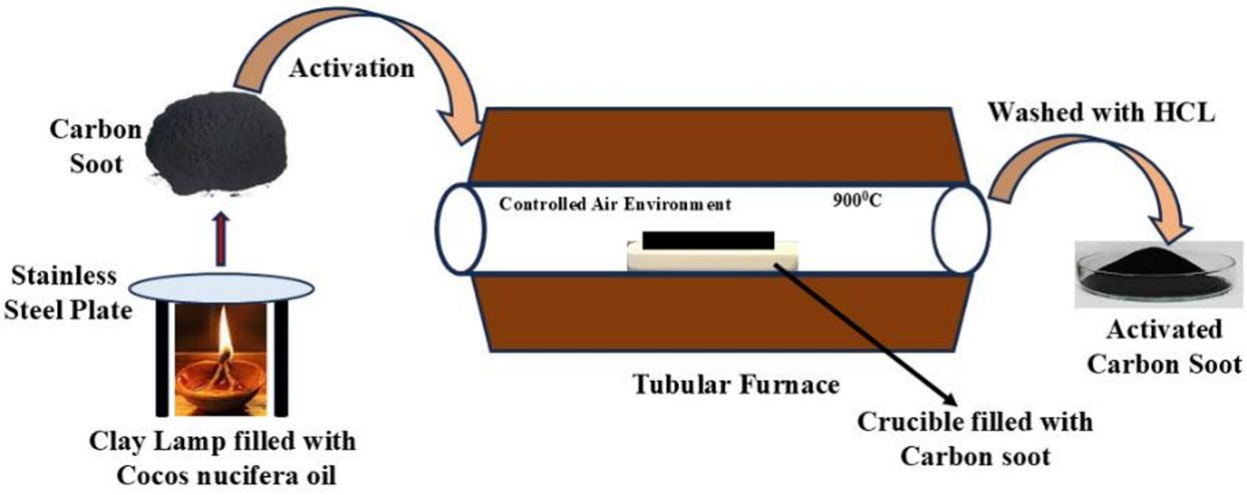
Schematic illustrate the synthesis pathway and activation mechanism of the prepared carbon soot.

In synthesis process clay lamp was filled with *Cocos nucifera* (Coconut Hair Oil) and burned with cotton wick in precise air environment by wick and oil process. The schematic representation of synthesis and activation process is shown in Fig. 2. During flame synthesis, mature soot particles were synthesized by different processes including pyrolysis, PHAs, soot nucleation, soot growth to soot oxidation as represented in Fig. 1.

In order to obtain synthesized material (carbon soot), a metal (stainless steel) plate was positioned above the clay lamp with the help of metal stand at a suitable height that flame touches the plate. The carbon soot that was produced was scraped off from the metal plate and stored in glass vial. The collected CS was further chemically treated by mixed with activating agent ZnCl_2_ and KOH in weight ratios, 1:1 and 1:2 respectively with mortar and pestle. The ratio of 1:1 for ZnCl_2_ and 1:2 for KOH with carbon soot and activation at 900◦C are chosen from our previous research^26–28^, to compare this research work with previous work done. Prepared mixtures were filled in crucibles and positioned in a programmable tubular furnace for thermal treatment at 900˚C under controlled air environment (i.e. to avoid gasification of carbon soot, crucibles were covered with the lid and restrict the airflow into the furnace) at 10^◦^C/min heating rate for 1 h. The compositions with CoCS: ZnCl_2_ as 1:1 and CoCS: KOH in 1:2 are named as CoACS_ZnCl2_ and CoACS_KOH_, respectively. The activating agent KOH is used for activation of carbon because of its property to produce highly porous network compared to other activating agents like ZnCl_2_ or H_3_PO_4_^29–31^. Additionally, due to the growth of -OH functional groups on the surface of the activated material surface area of carbon soot increases^32^. Prior research has already examined a range of carbon/KOH ratios, from 1:0.5 to 1:8 using various bio-precursors/carbon soaked with KOH or dry mixing. V. Sahu et al.^33^ use carbon soot damped with KOH in 1:7 ratio and show the maximum specific capacitance of 70 F g^− 1^ while S. Jung et al.^34^ used higher amount of KOH with carbon soot (i.e. 1:8) which shows the capacitance value 100 F g^− 1^. In this study, dry mixing was done using low wt% of KOH with CoCS i.e. 1:2, to reduce the hazards effect of KOH on the human body and adverse effect on the environment^35^.

During this activation phase, a negligible amount of CO_2_ and moisture were also added to the air. The chemical procedure involved between CoCS and KOH are as follows^36^:

*Stage 1*: 4 KOH + C (CoCS) = K_2_O + 2 H_2_ + K_2_CO_3_.

High temperatures cause KOH to react with amorphous CoCS produces K_2_CO_3_ and breakdown product K_2_O with hydrogen. This lowers the quality of CoCS when KOH decays into K_2_O. Additional reactions occur during CoCS activation, as indicated by following stages:

*Stage 2*: 2 KOH = K_2_O + H_2_O.

*Stage 3*: C (CoCS) + H_2_O (steam) = H_2_ + CO.

*Stage 4*: CO + H_2_O = H_2_ + CO_2_.

*Stage 5*: K_2_O + CO_2_ = K_2_CO_3_.

*Stage 6*: K_2_O + H_2_ = 2 K + H_2_O.

*Stage 7*: K_2_O + C (CoCS) = 2 K + CO.

*Stage 8*: K_2_CO_3_ + 2 C (CoCS) = 2 K + 3 CO.

During stage 2 steam is produced by the air’s moisture during thermal treatment and eliminates amorphous COCS, which becomes CO in stage 3 and enhance the porosity of CoCS. Additional carbon is also utilized for the conversion of K^+^ into K in stages 7 and 8. As the material develops pores, this extra carbon ceases to exist and lowering the yield. Enhancing the porosity with stages 1, 7 and 8 is one of the main advantages of employing KOH as an activating agent to activate the synthesized carbon soot in controlled air atmosphere^37–39^. Prepared material was washed many times using aqueous acid solution and DI water, which helps to remove K^+^ and other K, causing the stretched carbon structure to lose its previous shape. As a result, the porosity and surface area are improved. Generally, two stages of heat treatment are needed during the KOH activation process. Step one involves dehydration procedures for 0.5 to 1.5 h at 350 to 400 degrees Celsius, while step two involves activation for 0.5 to 5 h at high temperatures (750 to 900 degrees Celsius)^40,41^. In the current research, the CoCS: KOH mixture is heated to 900^◦^C in a single step to activate CoCS.

There is no single dominant stoichiometric redox reaction between carbon soot (CoCS) and ZnCl₂ like with KOH activation. Instead, ZnCl₂ interacts with carbon soot mainly through Lewis’s acid coordination, dehydration catalysis and molten-salt templating with only minor secondary reactions at high temperature. At low temperature (~ 290◦C) ZnCl_2_ melts and penetrates carbon soot. This weakens C–O and adjacent C–C bonds and promotes carbon framework rearrangement^42–45^. At higher temperature 800◦C-900◦C the carbothermal reduction may occur

1 $${\mathrm{ZnCl}}_{2}+\mathrm{C}\to\mathrm{Zn(g)}+{\mathrm{CCl}}_{x}$$

A significant amount of CoACS_ZnCl2_ and CoACS_KOH_ was collected after activation. Obtained activated carbon soot CoACS_ZnCl2_ and CoACS_KOH_ was treated with a 1 M HCl solution and washed with distilled (DI) water to bring the pH level down to neutral and dried in vacuum oven at 100◦C for 12 h.

### Characterization of the materials

The X-ray diffraction was performed using the PANalytical X′pert Pro model (Netherlands) to perform structural analysis of the activated CoCS_ZnCl2_, CoCS_KOH_ samples and raw CoCS. The power supply was set at 45 kV and 40 mA to operate and generate Cu-Kα radiation of wavelength λ = 0.154 nm, The scan was performed at a scan rate of 3˚ min^− 1^ between 10˚ and 90˚ (2θ). Touch LX2, Quantachome, (USA) was utilized to estimate the surface area and investigate the pore size and pore volume of a porous activated carbon. The surface morphology imaging of CoCSZnCl_2_ and CoCSKOH was photographed and recorded by using a scanning electron microscope (SEM) (Carl Zeiss, Model: Zeiss Gemini SEM, Germany) with an energy dispersive X-ray analysis (EDAX analyzer) function.

### EDLC fabrication and characterization

To create the electrode slurry, CoCS, conductive acetylene black, and binder PVdF-HFP were combined in a weight ratio of 7:2:1 in N-methyl pyrrolidone (NMP) solvent. The resulting homogenous slurry was distributed uniformly on polished flexible graphite sheets and dried for 12 h at the temperature of 80 °C to synthesize EDLC electrode. Each electrode (1 cm²) was loaded with approximately 0.96 mg of active material for device fabrication. The liquid electrolyte, 0.5 M NaPF_6_ in EC: PC (v/v), soaked Whatman filter sheets, was inserted between the two symmetrical electrodes to create the EDLC cells. CoCS_ZnCl2_ and CoCS_KOH_ were used as electrode materials to create the symmetric electric double layer capacitor (EDLC) cells. At ambient temperature, the liquid electrolyte shows an ionic conductivity of about 2 mS cm^− 1^. The electrochemical characteristics of the EDLCs were analysed using Zive Potentiostat (Zive SP1, WonATech Co. ltd., Korea) was used. The electrochemical impedance spectroscopy (EIS), galvanostatic charge-discharge (GCD) and cyclic voltammetry (CV) were also performed. CV was done in the potential range of 0–1 V. EIS investigations were carried out at a potential of 50 mV in the frequency range of 25 mHz to 1 MHz.

**Table 1:** Summary of precursor, activating agents, weight ratios (wt./wt.), and corresponding sample codes for coconut oil-derived carbon soot.

Precursor	Activating agent	Wt. %	Sample codes
Coconut oil	--	--	CoCS (Bare Soot)
	ZnCl_2_	1:1	CoACS_ZnCl2_
	KOH	1:2	CoACS_KOH_

## Results and discussion

### XRD studies

**Fig. 3:**
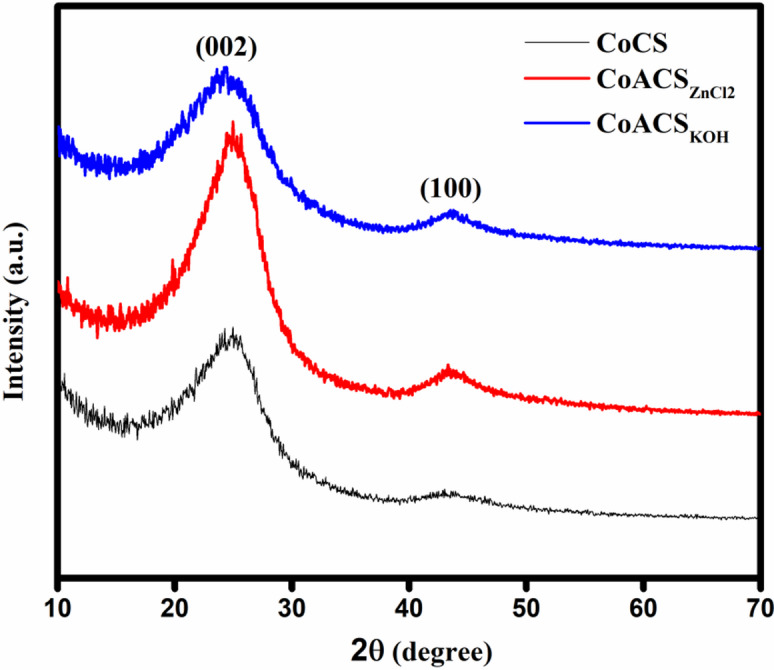
X-ray diffraction (XRD) patterns of CoCS (bare soot), CoACS_ZnCl₂_, and CoACS_KOH_.

Figure 3 depicts the XRD diffractograms of carbon soot synthesized using coconut oil named as CoCS, CoACS_ZnCl2_ and CoACS_KOH_. The observed intense diffraction peak at 2θ ~ 24.5º represent (002) graphitic planes while the peak at 2θ ~ 43.47º represent (100) plane respectively with reference to JCPDS card no. 41–1487.

The plane (002) indicates improved crystallite domains as activation was done at 900 °C while peak (100) represents the graphitic layers are not completely stacked and composed of turbostratic structures^46,47^. It can be seen from Fig. 3 that the crystallinity enhanced using ZnCl_2_, while using KOH peaks are broaden and reduced in height after the activation. CoACS_ZnCl2_ shows the greater peak at 2θ ~ 43.47º this specifies the enhanced alignment of the aromatic layers while CoCS and CoACS_KOH_ shows the disorderly orientation of graphitic planes as peaks at 2θ ~ 43.47º are shorter and wide. The crystallinity ($$\chi)$$of the carbon soot compositions is estimated using relation; $$\chi=\left(\raisebox{1ex}{${A}_{c}$}\!\left/\!\raisebox{-1ex}{${A}_{o}$}\right.\right)\times100\%$$, where $${A}_{c}$$is the area of the dominant crystalline peak and $${A}_{o}$$ is the area under whole diffractogram. The CoACS_KOH_ shows crystallinity 36% which is close to that of bare soot CoCS 33% and the composition activated with ZnCl_2_, i.e. CoACS_ZnCl2_, display higher crystallinity 47%.

The Bragg’s law is used to calculate inter-layer spacing (d-spacing), as indicated in Eq. 2, where d is the interplanar spacing, θ is the angle of diffraction, n is an integer and λ is wavelength of X-rays used.

2 $${\text{2d sin }}\theta {\text{ }} = {\text{ n}}\lambda$$

In CoCS, the maximum intensity peak is observed at 24.5˚ shown in the XRD pattern (Fig. 3) and displays d-spacing of 3.62 Å, slightly superior than the d-spacing of graphitic carbon (~ 3.4 Å)^48^. For CoACS_ZnCl2_ and CoACS_KOH_, d-spacing are marginally enhanced from 3.62 Å (~ 3.65 Å and ~ 3.71 Å respectively) after activation.

Equation 3 was used to estimate crystallite size using (002) diffraction plane (the strong peak), where t is the crystallite size, λ (Cu/K_α_ = 0.154 nm) is the wavelength of the X-rays, β (in radian) represents the full width at half maximum (FWHM) of XRD peak, θ (in radian) represents the Bragg’s angle, Scherrer’s constant k has the value 0.9 for spherical shape for soot agglomerates.

3 $$t=\frac{k\lambda}{\beta Cos\theta}$$

The crystallite size of CoCS ~ 1.92 nm which remains same after activation for CoACS_ZnCl2_ ~ 1.92 nm and CoACS_KOH_ shows crystallite sizes of ~ 1.40 nm. The enhanced d-spacing and reduced crystallite size of CoACS_KOH_ indicates that after activation with KOH, porosity and surface area may improve. The broad (002) reflection confirms a largely turbostratic carbon structure with limited long-range graphitic order. The crystallite size calculated using the Scherrer equation corresponds to the average coherent domain size rather than distinct crystalline graphite particles.

### SEM and EDX studies

**Fig. 4:**
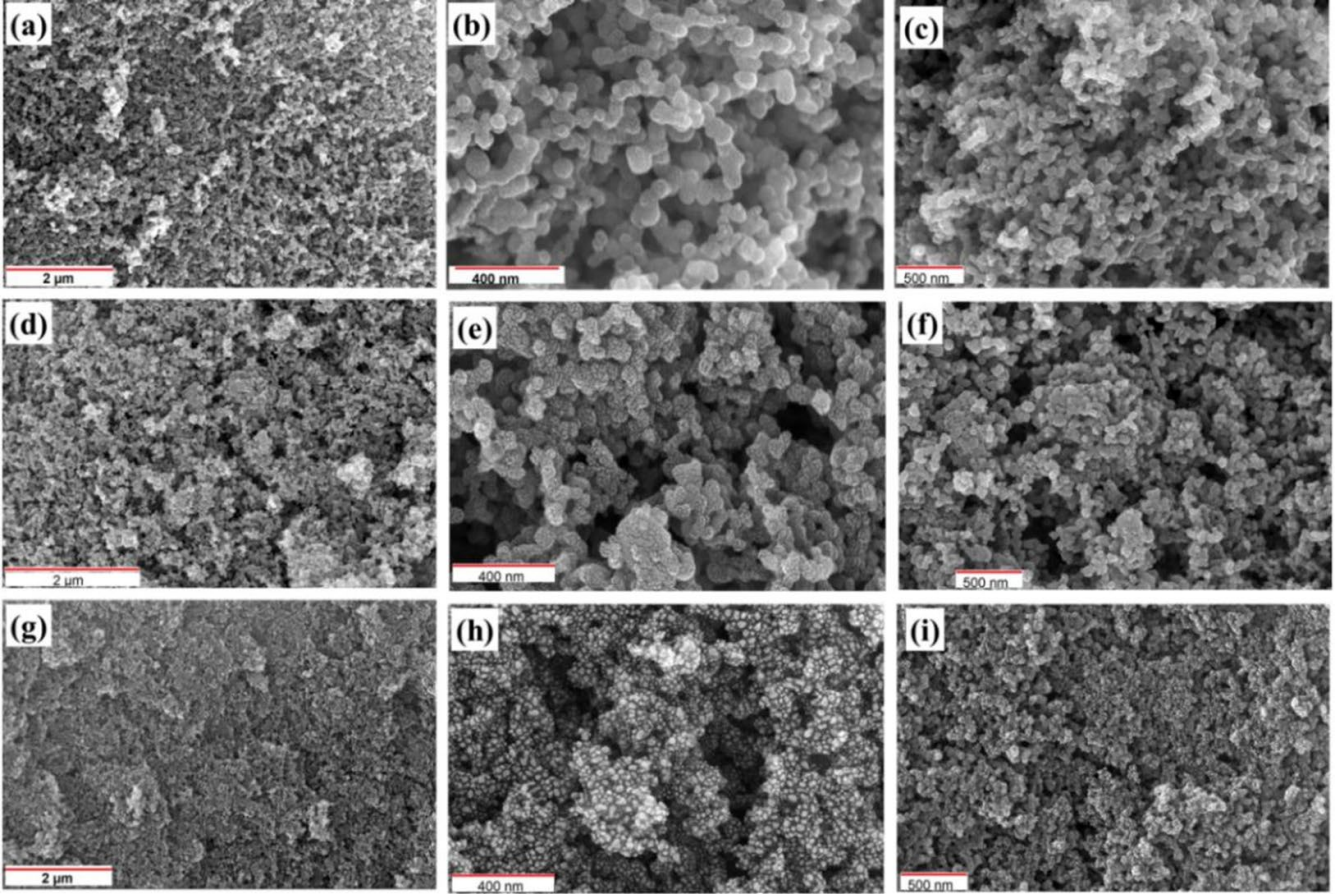
SEM images of coconut oil-derived carbon soot: **a**–**c** CoCS, **d**–**f** CoACS_ZnCl₂_, and **g**–**i** CoACS_KOH_ at varying magnifications.

The SEM micrographs of CoCS, CoCS_ZnCl2_ and CoCS_KOH_ at different levels of magnifications are represented in Fig. 4. All micrograph represents well interconnected micro porous network, which are analogous spongy structure of all prepared samples. CoCS (Fig. 4a-c) shows the spherical geometries, with well-connected and packed disordered agglomerations.

The particle size of these agglomerates varies between 50 and 60 nm. After the activation using activating agent ZnCl_2_ in 1:1 (wt. ratio) sample CoACS_ZnCl2_ (Fig. 4d-f) represents well packed fragmented clusters of agglomerations of size 30–35 nm. On the other hand, activation with KOH in 1:2 (wt. ratio) sample CoACS_KOH_ (Fig. 4g-i) shows well packed feather like agglomerates of particles size ~ 10–20 nm. SEM studies confirm the reduction in agglomeration particle size after activation and develop the porous structure that improve the specific surface area and enhance the electrochemical performance.

**Fig. 5:**
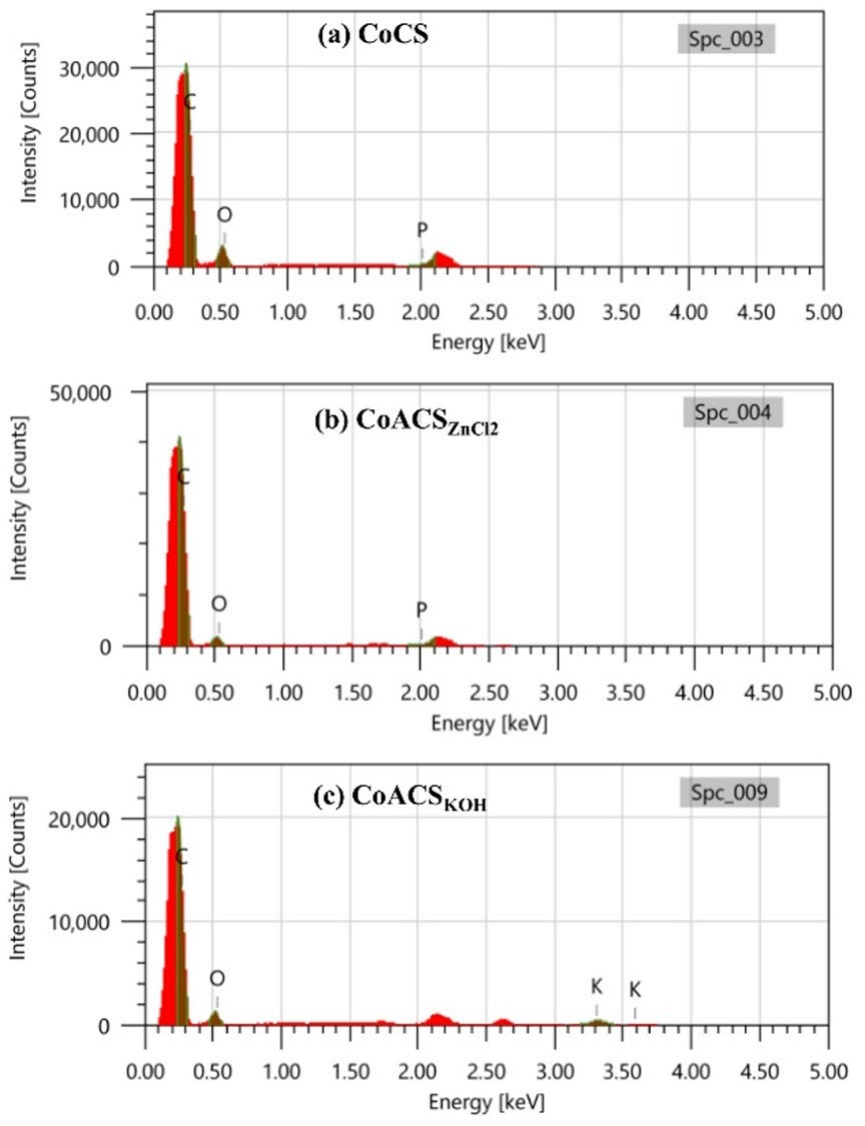
EDAX spectra of **a** CoCS, **b** CoACS_ZnCl2_ and **c** CoACS_KOH_, showing the elemental composition of the carbon soot.

Figure 5 represents the EDAX profile of coconut oil derived carbon CoCS, CoACS_ZnCl2_ and CoACS_KOH_. The elemental composition has been estimated using EDAX. It is very clear that in CoACS_ZnCl2_ and CoACS_KOH_, the carbon content increases after chemical and thermal treatment. The increase in carbon atomic percentage after ZnCl₂ activation is attributed to its role as a dehydrating and aromatizing agent during high-temperature treatment. ZnCl₂ facilitates the removal of heteroatoms (H, O and N) present in the carbon matrix and indorses condensation of carbon fragments into more stable fused aromatic (sp²) structures. It also helps in the conversion of aliphatic carbon into graphitic domains. As non-carbon elements are eliminated and the carbon framework becomes more ordered, the relative carbon content increases in CoACS_ZnCl₂_ compared to CoCS.

All samples contain the good amount of oxygen, with trace amount of potassium in CoACS_KOH_. The elemental compositions of all characterized samples (atomic and weight%) are shown in Table 2.

**Table 2:** Elemental composition (weight% and atomic %) of carbon (C), oxygen (O), and potassium (K) in CoCS, CoACS_ZnCl₂_, and CoACS_KOH_, as determined from EDAX analysis.

Element	CoCS		CoACS_ZnCl2_		CoACS_KOH_	
	Wt. %	At. %	Wt. %	At. %	Wt. %	At. %
C	56.94	63.78	71.52	76.98	61.84	69.68
O	43.06	36.22	28.48	23.02	34.23	28.96
K	--	--	--	--	3.93	1.36

### FTIR studies

**Fig. 6:**
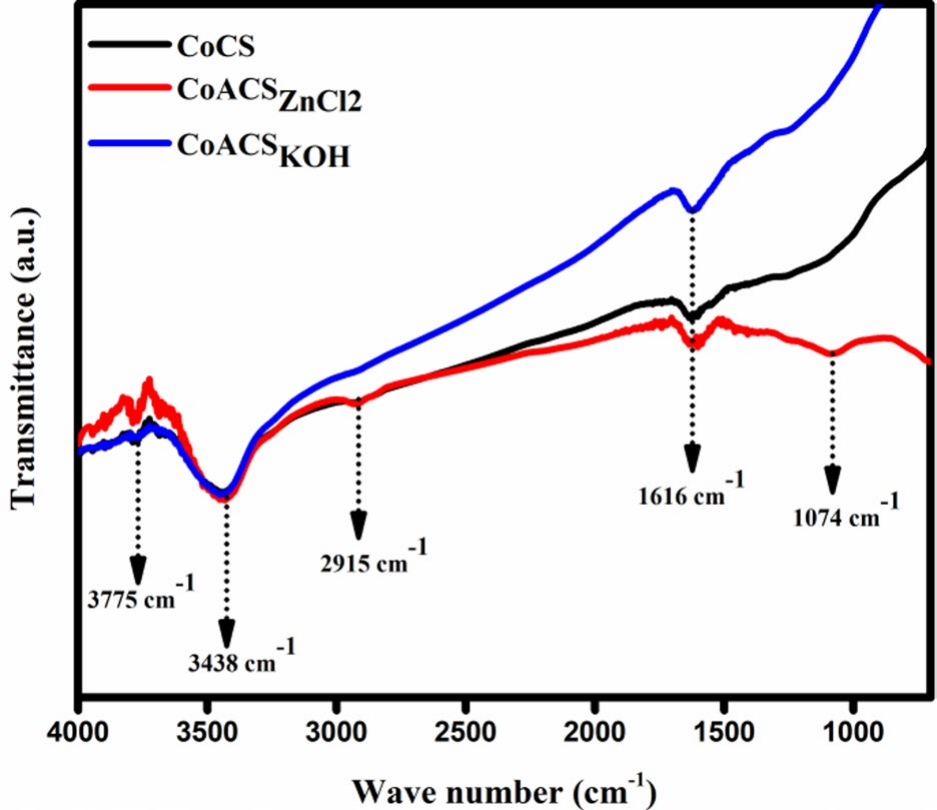
FTIR spectra of coconut oil-derived carbon soot before and after activation (CoCS, CoACS_ZnCl2_ and CoACS_KOH_).

FTIR spectra in 400–4000 cm^− 1^ region is shown in Fig. 6, to confirm the occurrence of distinct vibrational groups on the surface of CoCS, CoACS_ZnCl2_ and CoACS_KOH_.

The spectra band at 3775 cm^− 1^ represents the existence of O-H vibrations of free alcohol groups and the most prominent and broad peak at 3438 cm^− 1^ confirms the presence of intermolecular bonded alcohol (O-H stretching vibrations), showing the existence of water molecules in the prepared activated carbon soot. The peak at 2915 cm^− 1^ is attribute to the symmetrical and asymmetrical stretching vibrations of C-H bond. The peak at 1616 cm^− 1^ corresponds to the carbon-carbon bond (C = C) aromatic skeletal stretching, while 1074 cm^− 1^ appearances of the existence of C-O band.

**Table 3:** Characteristic FTIR absorption bands and corresponding functional group assignments for coconut oil-derived carbon soot (CoCS)^49–51^.

IR Spectrum Wavenumber	Functional Groups
1074 cm^− 1^	C-O stretching vibrations
1616 cm^− 1^	C = C aromatic skeletal stretching
2915 cm^− 1^	C-H symmetric and asymmetric bending
3775 cm^− 1^ and 3438 cm^− 1^	O-H stretching vibration in water molecules

The IR bands observed from FTIR spectra and their groups and compounds are listed in Table 3 and familiarize that after thermal treatment in air ambient atmosphere oxygen functionalities have been introduced.

### BET studies

**Fig. 7:**
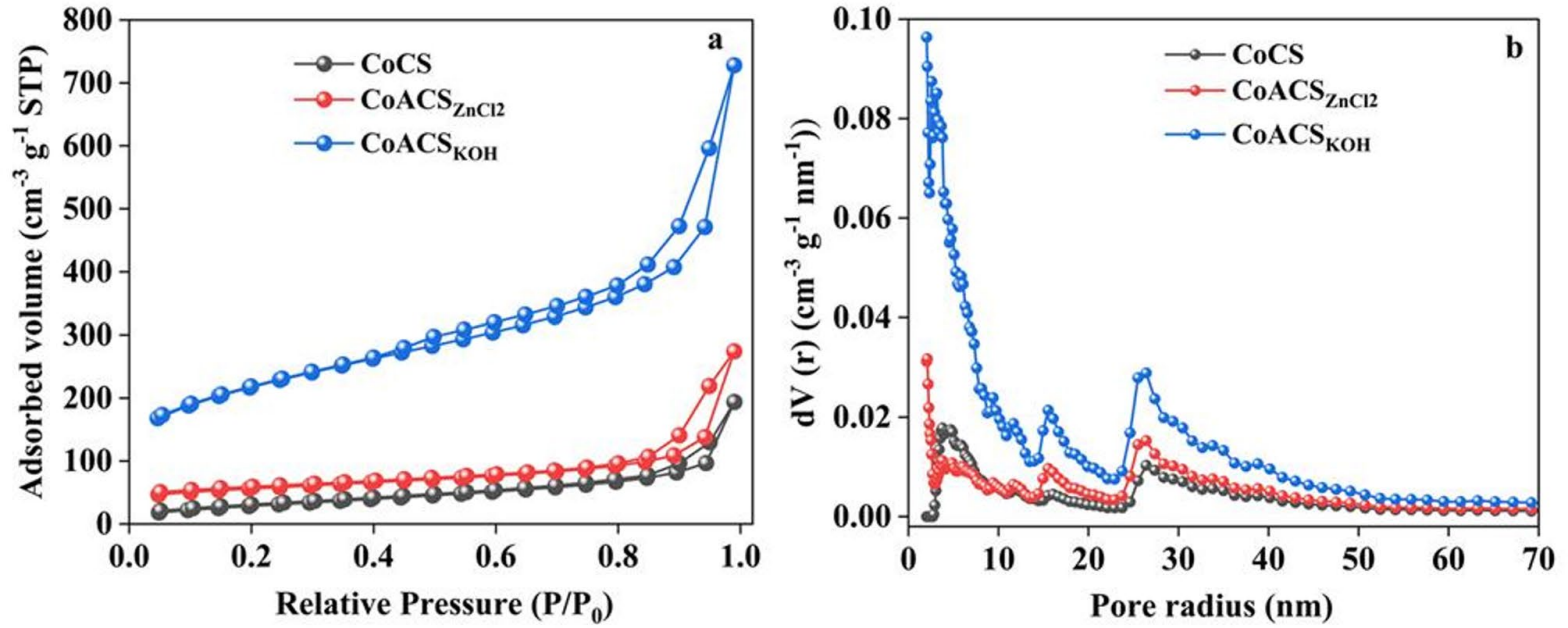
**a** Nitrogen adsorption–desorption isotherms and **b** pore size distribution curves of CoCS, CoACS_ZnCl₂_, and CoACS_KOH_.

BET adsorption-desorption isotherms were obtained using N_2_ estimate the specific surface area and pore characteristics of CoCS, CoCS_ZnCl2_ and CoCS_KOH_. Figure 7a exhibits the N_2_ adsorption-desorption graphs with respect to the relative presure^52^. In the relative pressure range 0.5–0.9, the isotherm signifies the capillary condensation and formation of multilayer. CoCS, CoCS_ZnCl2_ and CoCS_KOH_ type IV isotherm with H4 hysteresis curve. Enhanced adsorbed volume at P/P_0_ > 0.99, specifies the complex pore network in micro and meso-porous region in the material with narrow entrance. There is a sharp increment in the slop for CoCS_KOH_ at high relative pressure, this is associated with greater meso-pore volume with complex porous network^53,54^. BJH method was used to estimate pore radius of CoCS, CoCA_ZnCl2_ and CoCS_KOH_ and shown in Fig. 7b, illustrated the more meso-pores of radius ranging between 2 and 10 nm. In CoCS_KOH_, have larger pore volume than CoCS and CoCs_ZnCl2_ in meso-pore region with pore radius 4–6 nm. This indicates the growth of inter and intra clusters of pores after activation which offers the easy and smooth route for quick transportation of electrolytes ions^55,56^. Table 4 shows that the specific surface area of raw carbon soot CoCS is ~ 87.274 m^2^ g^− 1^ and increases after activation with activating agent ZnCl_2_ and KOH. CoCS_ZnCl2_ shows surface area ~ 207.690 m^2^ g^− 1^ and increases to ~ 742.774 m^2^ g^− 1^ for CoCS_KOH_. Improved surface area and meso porosity of activated CoCS_KOH_ are responsible to enhance electrochemical performance of energy storage devices^57^. This study confirms that CoCS_KOH_ can be a promising electrode material to improve the performance of EDLC devices.

**Table 4:** BET surface area and pore structure parameters, including specific surface area (S_BET_), total pore volume (V_total_), and average pore diameter for CoCS, CoACS_ZnCl₂_, and CoACS_KOH_.

Sample	S_BET_ (m^2^ g^− 1^)	V_total_ (cm^3^ g^− 1^)	Average Pore Diameter (nm)
CoCS	87.274	0.3006	1.074
CoCS _ZnCl2_	207.690	0.4250	8.341
CoCS _KOH_	742.774	1.1289	5.828

### XPS analysis

**Fig. 8:**
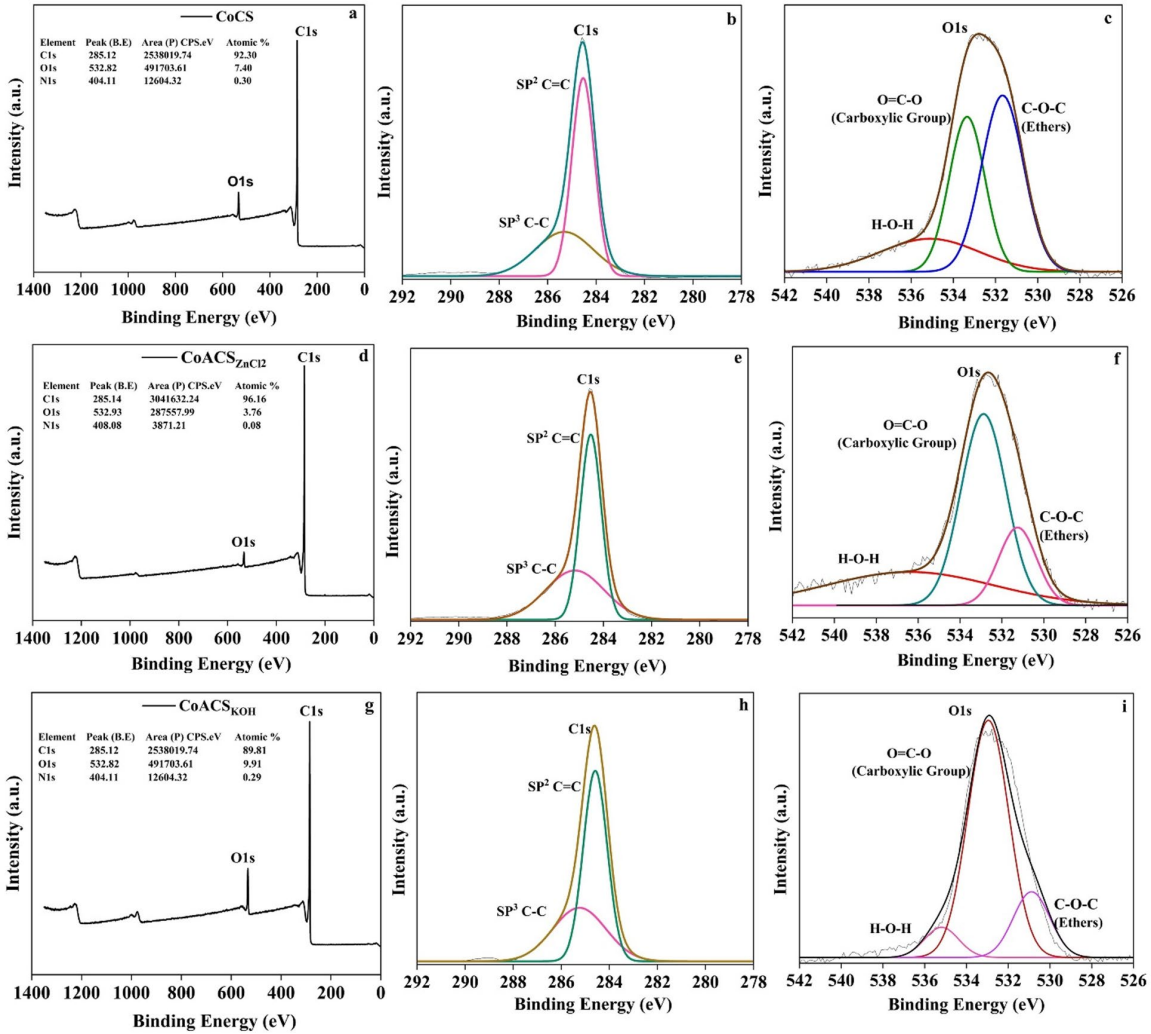
Full XPS survey spectra and high-resolution spectra for C1s and O1s of activated carbon samples; from left to right (**a**–**c**), (**d**–**f**), and (**g**–**i**) show the deconvoluted peaks for CoCS, CoACS_ZnCl₂_, and CoACS_KOH_.

The X-ray photoelectron spectroscopy (XPS) was used to analyze the elemental composition and chemical states of the elements present in CoCS, CoCS_ZnCl2_ and CoCS_KOH_. The complete XPS spectrum of CoCS, CoACS_ZnCl2_, and CoACS_KOH_, shown in Fig. 8. In Fig. 8a, d and g, the peaks associated to C1s and O1s are placed at binding energies (B.E) of ~ 285 eV and ~ 532 eV, respectively in all samples. Furthermore, the insets of figures provide the information about surface atomic compositions of C, O and N. CoACS_ZnCl2_ contains the higher carbon content ~ 96.16% than CoACS_KOH_ ~89.81%. CoCS and CoACS_KOH_ have the same amount of the nitrogen (atomic percentage ~ 0.3%) while CoACS_ZnCl2_ has least presence of nitrogen. Oxygen content in CoCS_KOH_ is more than the CoCS and CoCS_ZnCl2_ has minimum oxygen percentage.

The unrevealed C1s spectra of CoCS, CoCS_ZnCl2_ and CoCS_KOH_ shown in Fig. 8b, e and h, exhibit two prominent peaks at ~ 284 eV and ~ 285 eV, corresponding to sp² (C = C) and sp³ (C-C) hybridization of carbon atoms respectively, with similar binding energies across all samples. The presence of carbon-carbon bonds (C = C and C–C) contribute to enhanced electrical conductivity of carbon-based materials. The calculated sp² C = C/sp³ C-C ratios for CoCS, CoCS_ZnCl2_ and CoACS_KOH_ are 0.39%, 0.36% and 0.45%, respectively. Activation with KOH shows higher C = C (sp²)/C–C (sp³) ratio compared to the ZnCl₂ activated CoCS. This is likely due to enhanced aromatization, increased graphitic carbon content and structural reorganization. Which favoring the formation of more conjugated carbon domains suggests improved electron transfer kinetics, thereby enhancing electrical conductivity and electrochemical performance. Further analysis of the O1s spectra Fig. 8c, f and i reveals three distinct oxygen species. The deconvoluted peak at 531 eV is attributed to C–O–C (ether) functionalities, while the peaks at 533 eV and 535 eV correspond to O–C = O (carboxylic groups) and physiosorbed H₂O on the surface, respectively^58–61^.

### Galvanostatic charge- discharge (GCD) studies of the EDLCs

**Fig. 9:**
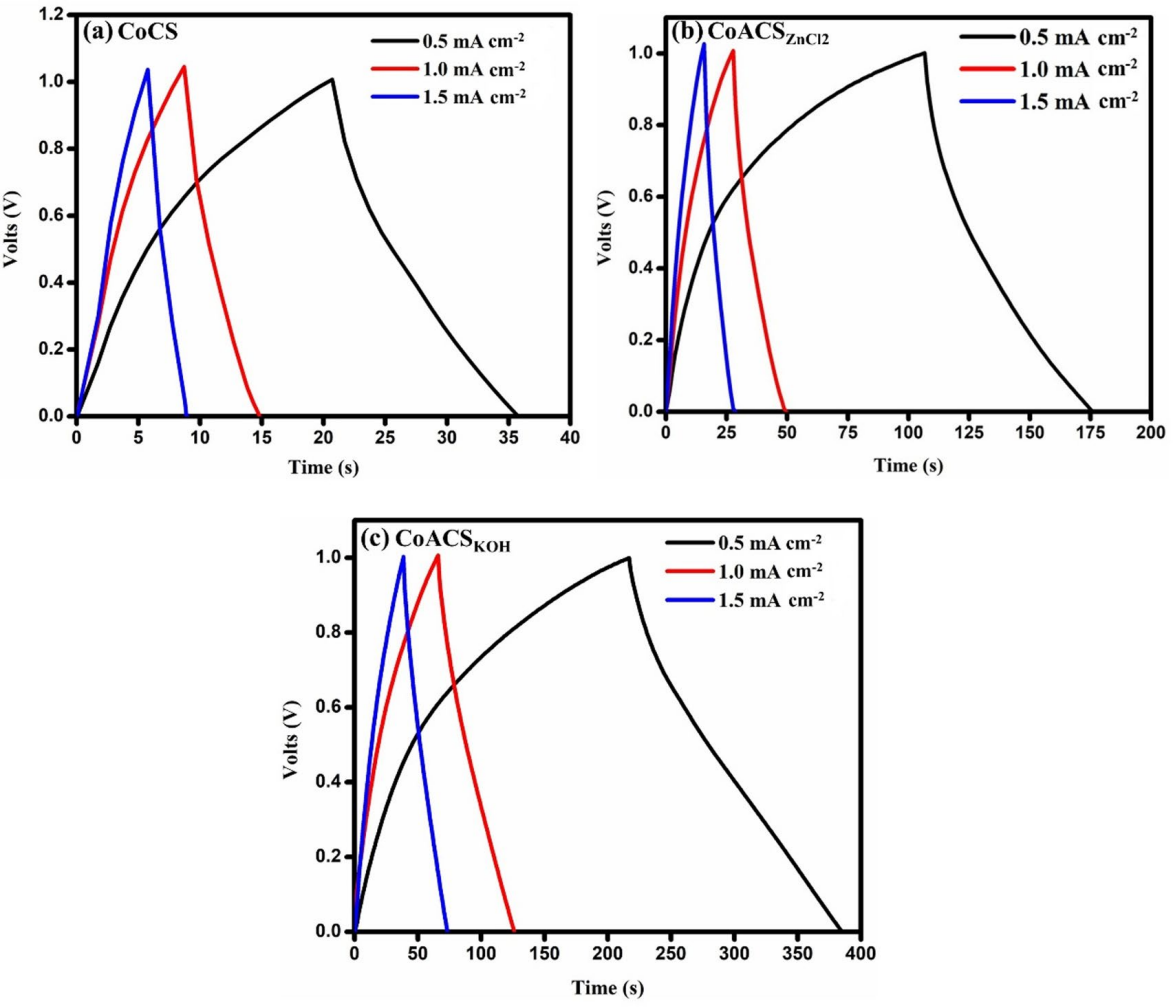
GCD plots of prepared coconut oil samples at different current densities; **a** CoCS, **b** CoACS_ZnCl2_ and **c** CoACS_KOH_, respectively.

For the examination of charging and discharging of EDLC, galvanostatic charge-discharge (GCD) characteristics were executed at different values of current densities. The GCD profiles are shown in Fig. 9. The geometry of all GCD plots is nearly triangular in shape and confirms the EDLC characteristics. While charging and discharging there is a small drop in initial voltage is observed, which specifies the internal resistance of material^62^. The ohmic loss restricts the access of porous network of electrode material by electrolyte ions and it is observed in all three EDLCs (Fig. 9a-c). The ohmic drop in coconut oil derived activated carbon, CoCS_KOH_ is ~ 0.004 V (Fig. 9c), which is lesser than earlier reported the mustard oil (0.09 V)^26^ and sesame oil (0.025 V)^28^ derived activated carbon. It confirms that the coconut oil derived electrode material offers lower resistance to electrolyte ions for facile motion into porous network.

The calculation of specific capacitance C_s_ was done using Eq. 4, while the specific energy density (${E}_{\mathrm{s}}$) and power density ($P$) were calculated using the Eqs. 5 and 6. Figure 9a confirms that the CoCS has the least discharging time, which corresponds to the specific capacitance ~ 16 F g^− 1^ at current density 0.5 mA cm^− 2^.

The GCD profiles of CoACS_ZnCl2_ (Fig. 9b) and CoACS_KOH_ (Fig. 9c) exhibit longer discharge times, indicating improved capacitive performance. CoACS_KOH_ is more capacitive having capacitance ~ 176 F g^− 1^ than CoAC_ZnCl2_ ~65 F g^− 1^ at current density 0.5 mA cm^− 2^. It is confirmed by the previous studies that the wt% of activating agent KOH has imperious involvement in activation process and plays key role in creating the micro porous structure^38^. This demonstrates that the ideal optimum ratio, within the comparative framework of this study, of chemical activation in this work is 1:2 wt% ratio of coconut oil derived carbon soot with KOH. The estimation of specific capacitance (C_s_) for all prepared devices was done using Eq. 4. where m represents the mass of active material loaded of on single electrode in gram, I show the current (A), Δ t is the discharging time (s), and Δ V is the potential change (V).

 

4 $${\mathrm{C}}_{{\mathrm{s}}} {\text{ = 2 }} \times \frac{I{\Delta}\mathrm{t}}{m{\Delta}\mathrm{V}}$$

The energy density E_s_ of the EDLCs was calculated using Eq. 5. where C_s_ is the specific capacitance and V is the potential.

 

5 $${\mathrm{E}}_{{\mathrm{s}}} {\text{ = }}\frac{1}{2}C_{s} V^{2} \times \frac{1}{{3.6}}$$

The power density of electrode was estimated using the following Eq. 6. E_s_ is energy density and Δt is the discharging time.

6 $${\mathrm{P}} = \frac{{E}_{s}}{\varDelta t} \times 3600$$

**Fig. 10:**
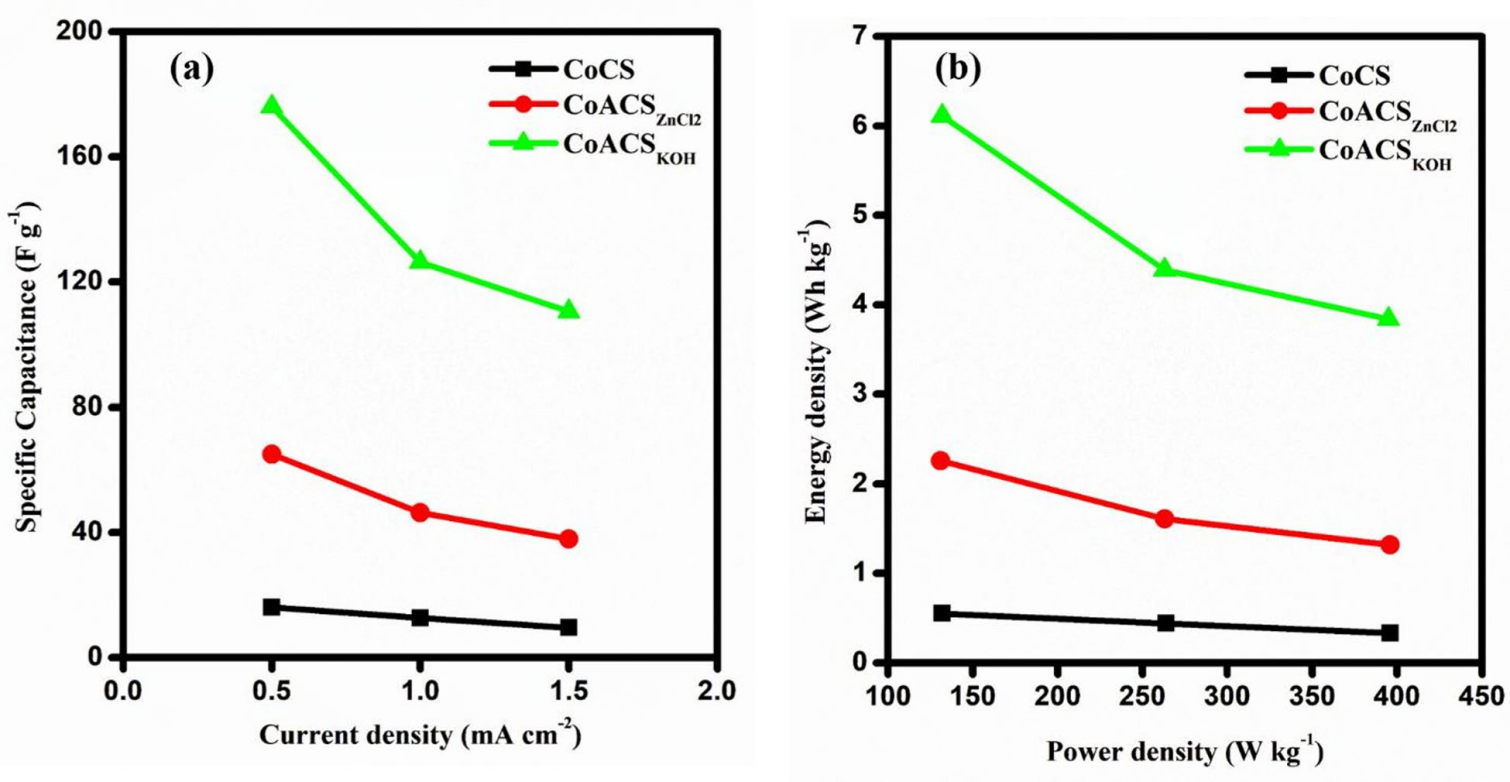
**a** Variation of specific capacitance with current density and **b** Ragone plot (energy density vs. power density) for symmetric EDLCs with CoCS, CoACS_ZnCl2_ and CoACS_KOH_ electrodes.

Figure 10a represents the variation in specific capacitance at different current densities. It can be observed that the specific capacitance decreases with respect to rise in the discharging current. The decrease in specific capacitance may be because the electrolyte ions do not completely fill the internal porous structure of the electrode material at higher scan rates^63^. The Ragone plots of coconut oil derived carbon soot are depicted in Fig. 10b. A significant increment in CoACS_KOH_ energy and power density due to activation can be observed.

In addition to rise in the power density, the constructed EDLC cell also faces decrement in specific energy density. For CoCS device specific energy density drops from ~ 0.55 Wh kg^− 1^ to ~ 0.33 Wh kg^− 1^ with the increase in the power density from ~ 132 W kg^− 1^ to 396 W kg^− 1^, while the specific energy density of the CoACS_ZnCl2_ device drops from 2.26 Wh kg^− 1^ to 1.32 Wh kg^− 1^ as the power density rise. The EDLC device prepared using CoACS_KOH_ shows maximum energy density ~ 6.11 Wh kg^− 1^ which reduces to ~ 3.84 Wh kg^− 1^. This explanation indicates that CoACS_KOH_ holds more energy towards higher values of power than other samples synthesized from coconut oil. The increased specific energy density in CoACS_KOH_, assigned to the easy and facile transportation of electrolyte ions into the double layer formed due to the porous structure. The pseudo effect in capacitance may associated with the occurrence of vibrational functional groups in electrode material^64,65^.

**Fig. 11:**
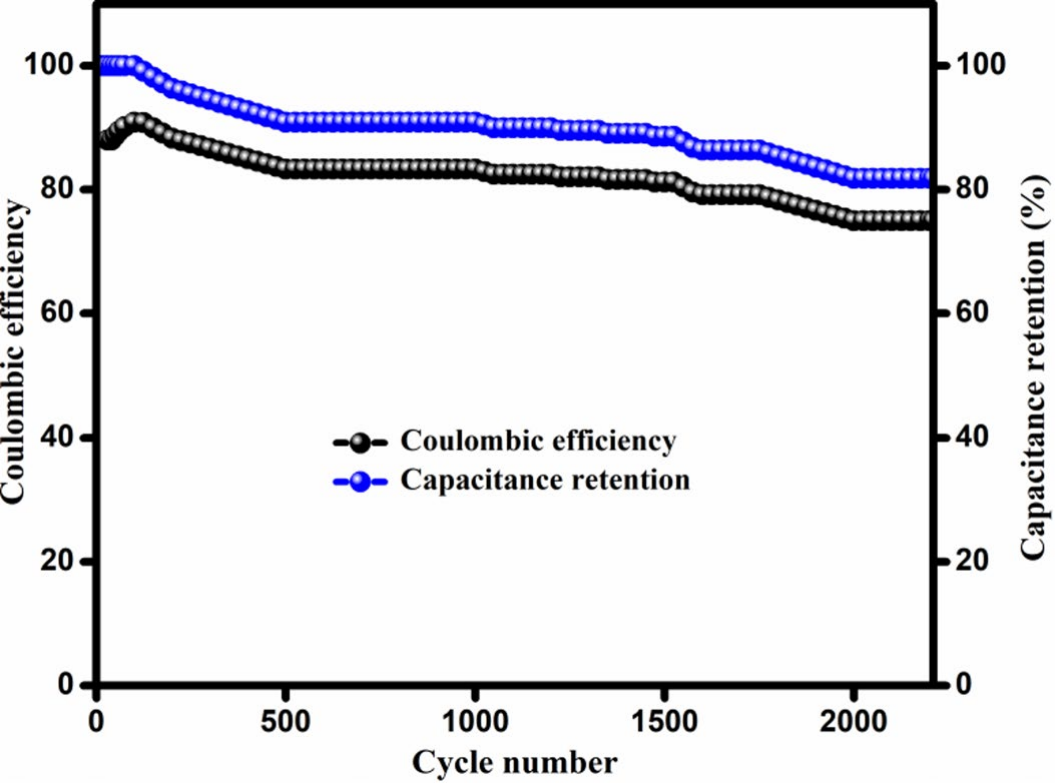
Cycling performance of CoACS_KOH_ symmetric device showing variation of capacitance retention and coulombic efficiency as a function of cycle number.

The variation in columbic efficiency and capacitance retention with cycle number is shown in Fig. 11. Cyclability is an essential aspect in the performance of energy storage devices. The examination of cycle life and the estimation of columbic efficiency of CoACS_KOH_ were completed by uninterruptedly charging/discharging at current density of 2 Ag^− 1^ using Eq. 7. After 2210 cycles of continuous charging/discharging, CoACS_KOH_ shows the capacitance retention ~ 76% with the coulombic efficiency more than ~ 80%.

### Cyclic Voltammetry (CV) studies

**Fig. 12:**
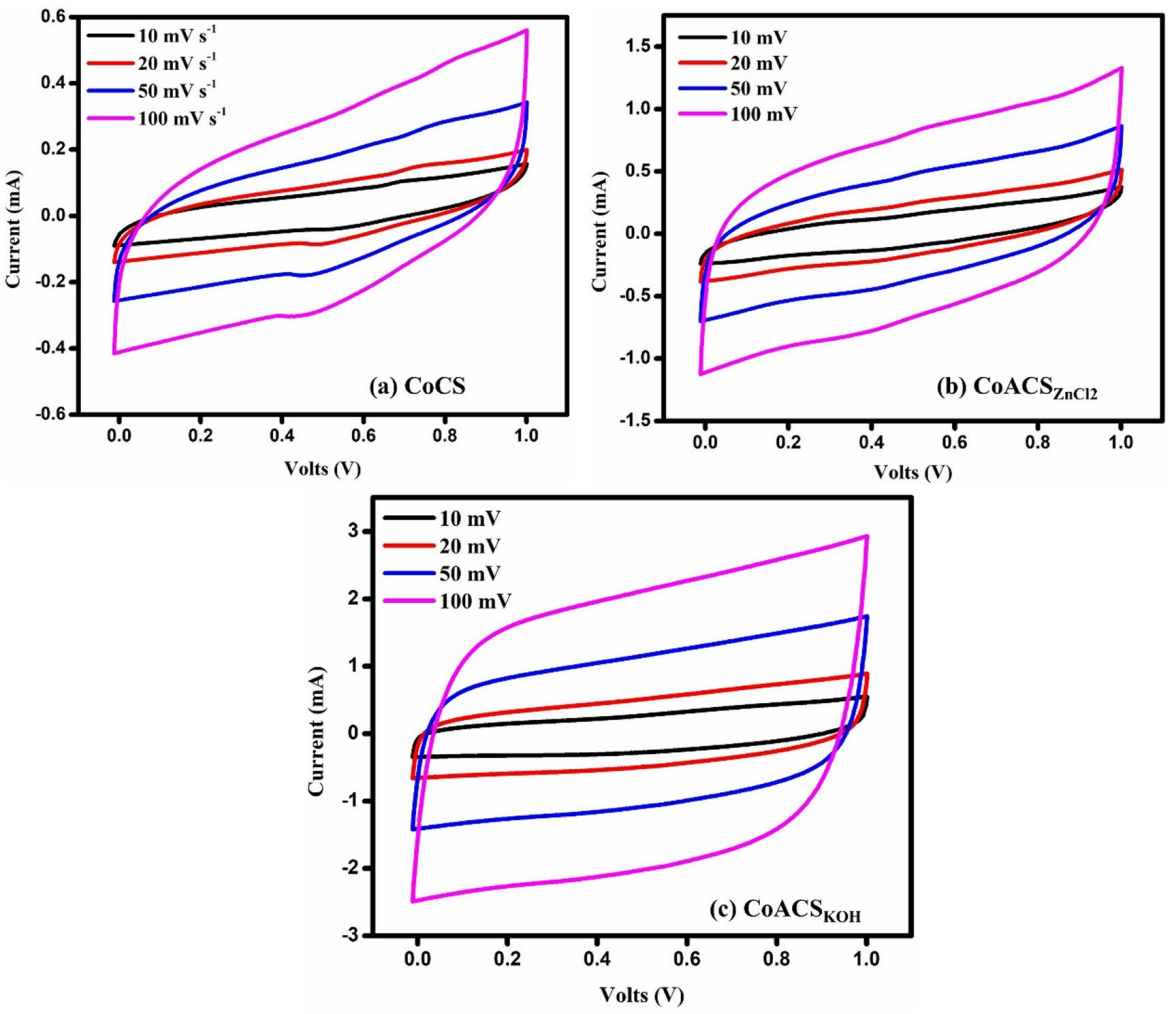
CV plots of **a** CoCS, **b** CoACS_ZnCl2_ and **c** CoACS_KOH_ respectively, different scan rates.

Figure 12 shows CV curves taken at different scan rates ranging from 10 mV s^− 1^ to 50 mV s^− 1^, with the potential window set to 0–1 V. All CV profiles are almost rectangular in shape and follow the EDLC charging and discharging patterns. Among the samples, CoACS–KOH displays a more pronounced rectangular shape, indicating improved capacitive performance and better charge storage characteristics. At higher scan rate, there is insignificant change in the CV curve profile from the standard rectangular shape, may ascribed the distributed charge storage which is a conventional behavior of porous electrode material^66,67^. This reveals that the CoACS_KOH_ is capable of reversibly shifting polarizations and has superior capacitive characteristics. Keeping the superior rectangular profile up to higher scan rates (50 mV s^− 1^) in CoACS_KOH_ discloses the enhanced rate competency of the supercapacitor interface between CoACS_KOH_ electrodes and the electrolyte compared to other EDLCs. This is due to the existence of a well-structured and inter-connected porous network in CoACS_KOH_ after activation. Equation 7 was used to compute specific capacitance (${C}_{s}$) at various scan rates. CoACS_KOH_ has a maximum specific capacitance of 55.41 F g^− 1^ at a scan rate of 10 mV s^− 1^.

7 $${C}_{s}=\frac{A}{2m\times s\times\varDelta V}$$

where A = integrated area under the CV curve (A·V), m = total mass of active material in both electrodes (g), s = scan rate (V s⁻¹) and ΔV = potential window (V).

**Fig. 13:**
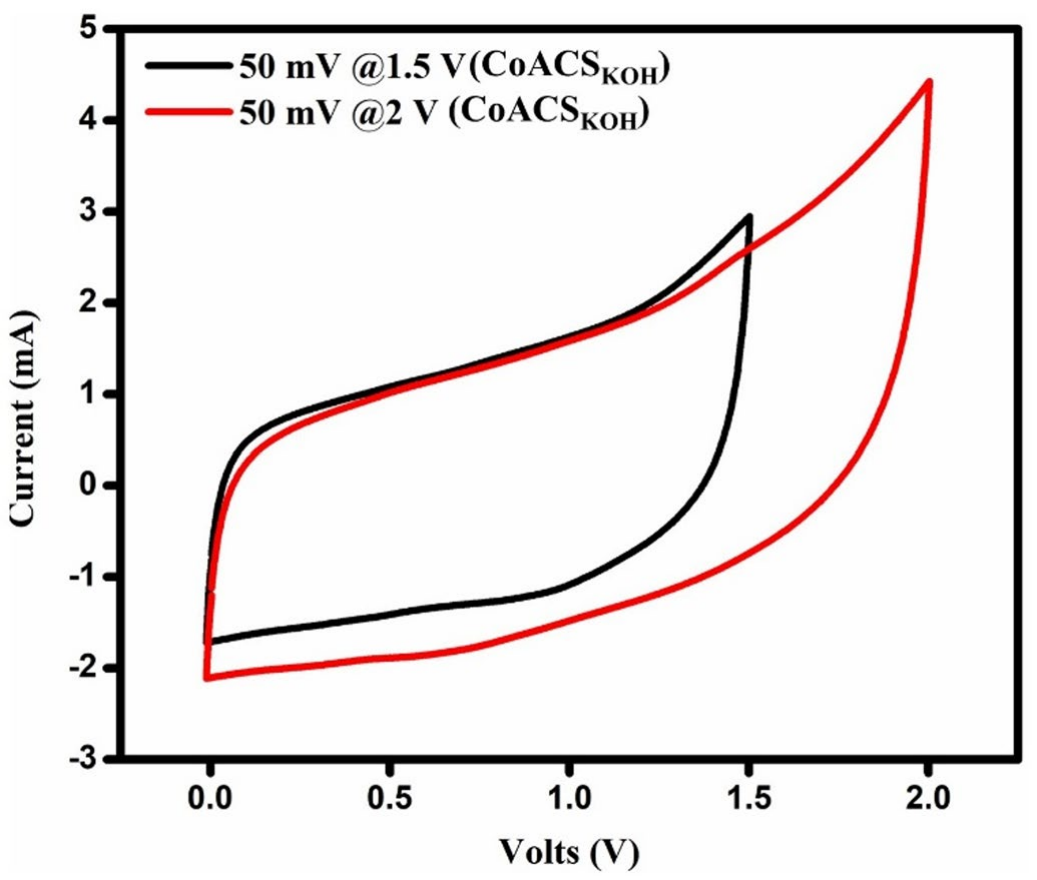
CV plots of CoACS_KOH_ sample in the wider potential window of 0–1.5 V and 0–2 V at 50 mV s^− 1^.

Figure 13 shows the CV plots of the CoACS_KOH_ device plotted at a scan rate of 50 mV s^− 1^ in the wider voltage window of 0–1.5 V and 0–2 V to better understand the relative parameters and properties of the EDLCs.

It is shown that all EDLCs have a roughly rectangular shape, which indicates better capacitive behavior. However, the current scale of CoACS_KOH_ is superior to other samples. It may be because of the better specific surface area given by CoACS_KOH_ for the creation of the double layer and the superficial migration of electrolyte ions towards the electrode-electrolyte interface.

### EIS studies of CoCS, CoACS_ZnCl2_ and CoACS_KOH_

**Fig. 14:**
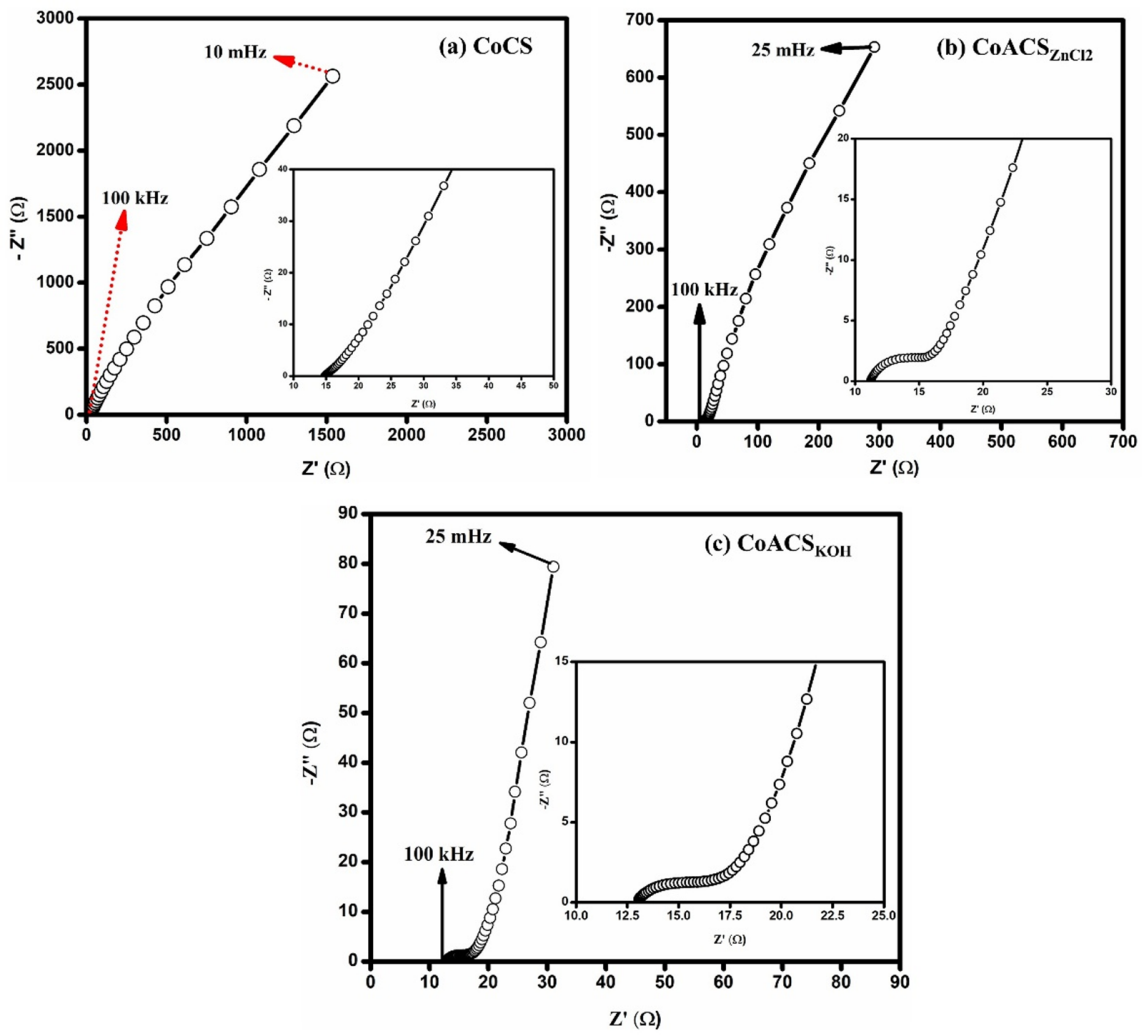
Impedance analysis of coconut oil derived carbon soot CoCS, CoACS_ZnCl2_, and CoACS_KOH_ in 0.5 M NaPF_6_ electrolyte.

Figure 14 depicts the typical nyquist plots from the EIS investigations. EIS studies displays the resistive and capacitive behaviour of carbon soot (electrode material) with respect to frequency. The EDLC device shows resistive nature towards higher frequencies and become capacitive at lower frequencies^68,69^. CoCS, CoACS_ZnCl2_ and CoACS_KOH_ Nyquist plots show a semicircle at high frequency that gradually grows towards the lower frequency side, corresponding to diffusion impedence and charge transfer impedence at electrode-electrolyte interface. The bulk resistance also occurs at high frequencies and estimated by the point of intersection at x-axis. At lower frequencies, the capacitive nature is represented by a vertical line parallel to y-axis since the electrolyte ions have lower diffusion resistance. Figure 14a, displays that the semicircle is absent, which demonstrate the ohmic connection of CoCS with the graphite sheet^64,70^.

The CoCS (Fig. 14a) shows bulk resistance ~ 14.8 Ω cm^2^, while CoACS_ZnCl2_ (Fig. 14b) and CoACS_KOH_ (Fig. 14c) show the bulk electrolyte resistance ~ 11 Ω cm^2^ and 12.7 Ω cm^2^, respectively. This indicates the easy access of electrolyte ions into the porous network after activation. For ideal capacitors the Nyquist plot must be parallel to imaginary axis at low frequencies. In this study, the imaginary component (Z″) shows a sharp rise and forms an almost vertical line in the low-frequency region, particularly for CoACS_KOH_. This suggests that CoACS_KOH_ exhibits more ideal capacitive behavior compared to CoACS_ZnCl₂_ and CoCS.

The above studies indicate that the electric double-layer (EDL) capacitance is the main charge storage mechanism in the system. However, a small contribution from distributed charge storage may have its own role. This additional effect can be corroborated to the porous structure of the material supporting efficient ion movement and surface interaction, in agreement with the CV results discussed earlier in the section "Cyclic Voltammetry (CV) studies".

From Fig. 14c, COACS_KOH_ represents the Nyquist curve is most parallel to y-axis towards lower frequency region e.g., COACS_KOH_ is more capacitive than CoACS_ZnCl2_ and CoCS, which is also confirmed from GCD and CV analysis.

## Conclusion

In this study, *Cocos nucifera* (coconut hair oil)-derived carbon soot via a simple and conventional flame synthesis (wick-and-oil) method was successfully prepared and evaluated as an electrode material for EDLCs using NaPF₆-salt based non-aqueous electrolyte. The obtained carbon soot exhibited a distinctive spherical, layered 3D onion-like morphology and the favorable structural parameters, with activation leading to enhanced porosity and improved electrochemical performance in EDLC. Between the two activating agents studied, KOH activation resulted in better electrochemical behavior compared to ZnCl₂ under the same conditions. The KOH-activated carbon delivered a specific capacitance of ~ 176 F g⁻¹, along with an energy density of ~ 6.11 Wh kg⁻¹ and a power density of ~ 395 W kg⁻¹. As presented in Table 5, the performance of the developed material is comparable to several reported KOH-activated carbons derived from different bio-precursors, as well as conventional carbon materials such as CNTs and graphene-based systems. The facile synthesis approach, the use of a widely available and low-cost precursor, and the promising electrochemical characteristics demonstrate a significant potential of *Cocos nucifera*-derived carbon soot as a sustainable, scalable, and high-performance electrode material for EDLC devices. Overall, the economic viability of the material is supported by the straightforward flame synthesis method and the potential use of non-edible or expired coconut oil as a precursor.

**Table 5:** Comparison of specific surface area (SSA), pore volume (V_Total_), and specific capacitance of selected KOH-activated carbon materials from different precursors, including the material developed in this study.

Carbon precursor	SSA (m² g⁻¹)	V_Total_ (cm³ g⁻¹)	Specific capacitance (F g⁻¹)	Reference
KOH-activated CNTs	2540	1.437	97	^71^
KOH-activated carbon/graphene nanosheets composite	2807	1.562	205	^72^
KOH-activated waste coffee ground	1355	0.167	105.3	^73^
KOH-activated Musa Acuminata stem	3351	1.99	102	^74^
KOH-activated Waste Termite Biomass	1440.650	0.4512	91.76	^75^
KOH Activated CoCS	742.774	1.289	176	In this work

## Data Availability

The datasets used and/or analysed during the current study available from the corresponding author on reasonable request.
